# Author Correction: Unequal exchange of labour in the world economy

**DOI:** 10.1038/s41467-026-77488-y

**Published:** 2026-09-04

**Authors:** Jason Hickel, Morena Hanbury Lemos, Felix Barbour

**Affiliations:** 1 Institute for Environmental Science and Technology (ICTA-UAB), Autonomous University of Barcelona, Barcelona, Spain; 2 Department of Anthropology, Autonomous University of Barcelona, Barcelona, Spain; 3 International Inequalities Institute, London School of Economics and Political Science, London, United Kingdom; 4 Stockholm Resilience Centre, Stockholm University, Stockholm, Sweden; 5 Beijer Institute of Ecological Economics, The Royal Swedish Academy of Sciences, Stockholm, Sweden

**Keywords:** Developing world, Sociology

Correction to: *Nature Communications* https://doi.org/10.1038/s41467-024-49687-y, published online 29 July 2024

In the version of this article initially published, the monetary time-series-related results were presented in current Euros, not constant Euros. The results have been updated to constant Euros in the HTML and PDF versions of the article. For comparison, the original manuscript is available as Supplementary Information accompanying this article.

## Supplementary information

Original, uncorrected article

