# Supplementary material for: Unequal exchange of labour in the world economy
Source: Nat Commun. 2024 Jul 29;15:6298. doi: 10.1038/s41467-024-49687-y (PMC11286830; doi:10.1038/s41467-024-49687-y)
Supplement: Supplementary file 1 — Supplementary Information [file 41467_2024_49687_MOESM1_ESM.pdf]

**SUPPLEMENTARY INFORMATION**

# Unequal exchange of labour in the world economy

Jason Hickel[1,2,3*], Morena Hanbury Lemos[1], Felix Barbour[4,5]

1. Institute for Environmental Science and Technology (ICTA-UAB), Autonomous University of Barcelona
2. Department of Anthropology, Autonomous University of Barcelona
3. International Inequalities Institute, London School of Economics and Political Science
4. Stockholm Resilience Centre, Stockholm University
5. Beijer Institute of Ecological Economics, The Royal Swedish Academy of Sciences

*Corresponding author: j.e.hickel@lse.ac.uk

## Supplementary Figures

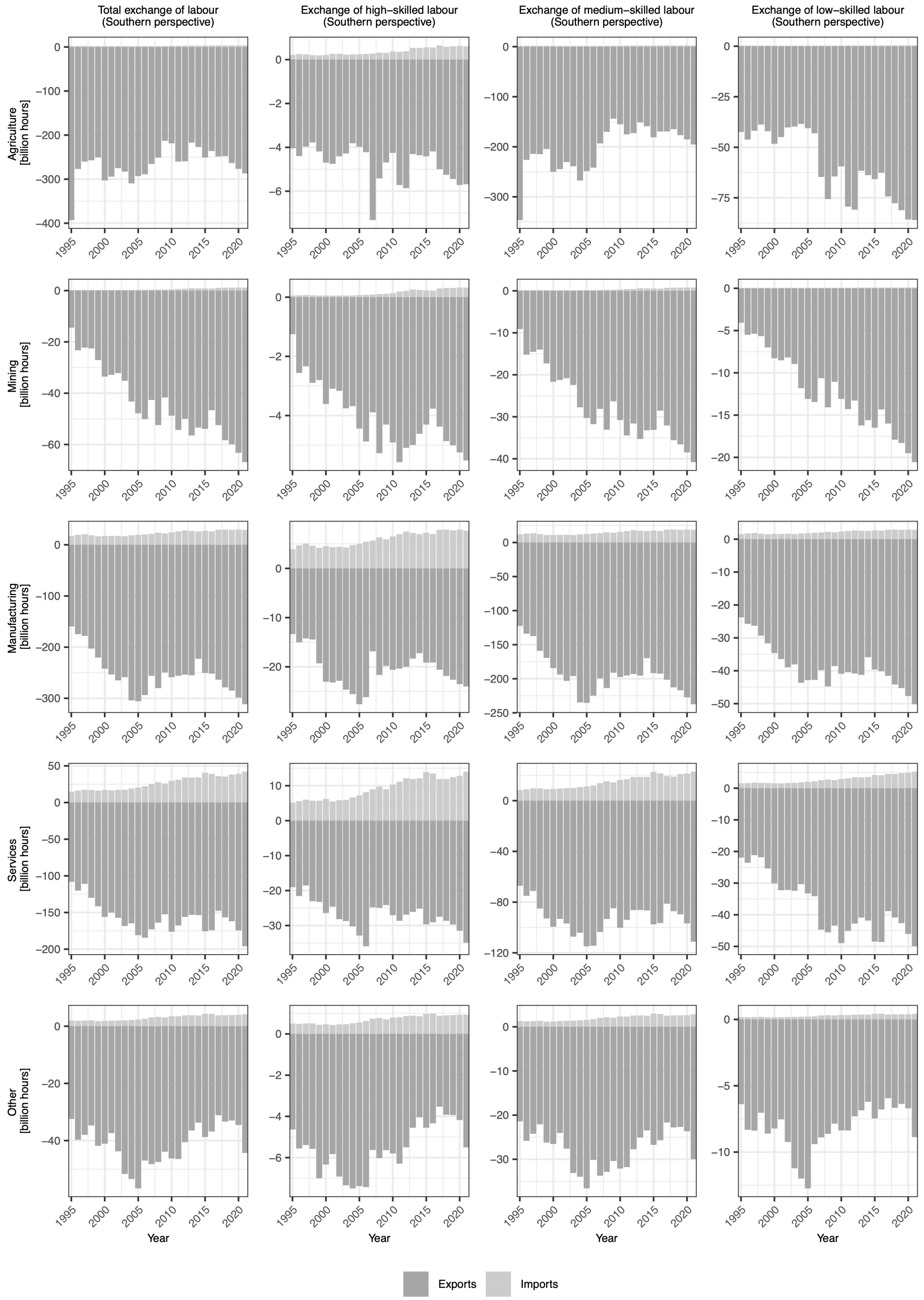


**Supplementary Fig 1.** Exports and imports of embodied labour (hours) by the global South, 1995-2021, by skill level within each sector.

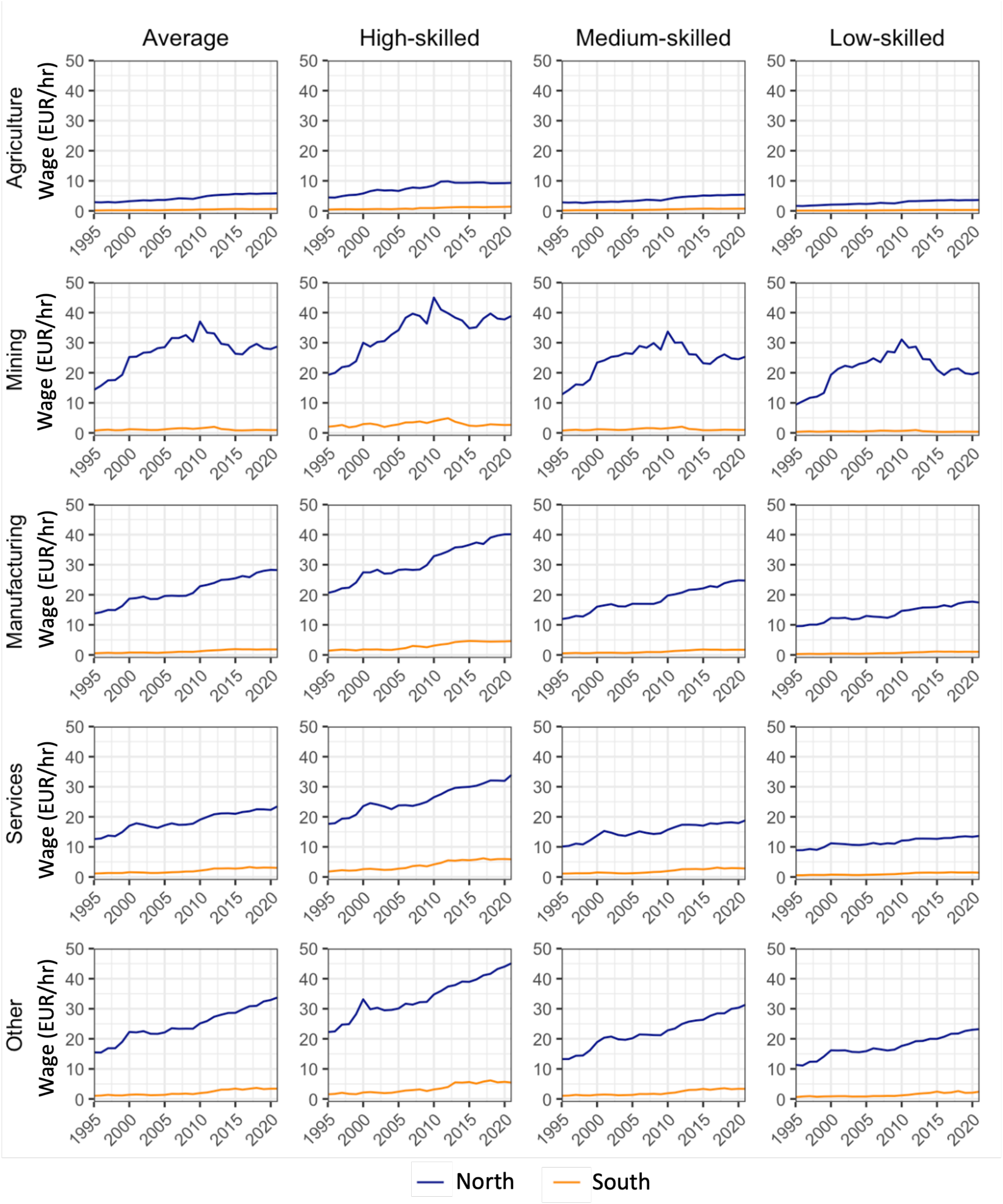


**Supplementary Fig 2.** Wage trends in the global North and South by skill level and sector, Euros per hour (constant 2005), 1995-2021.

**Supplementary Tables**

**ST 1.** Regional aggregations.

| **Global North / Core** | **Global South / Periphery** |
|---|---|
| Austria | Bulgaria |
| Belgium | Croatia |
| Cyprus | Hungary |
| Czech Republic | Lithuania |
| Germany | Poland |
| Denmark | Romania |
| Estonia | China |
| Spain | Brazil |
| France | India |
| Greece | Mexico |
| Ireland | Russia |
| Italy | Turkey |
| Luxembourg | Indonesia |
| Latvia | South Africa |
| Malta | |
| Netherlands | **Regional aggregations** |
| Portugal | Rest of Asia and Pacific |
| Sweden | Rest of America |
| Slovenia | Rest of Europe |
| Slovakia | Rest of Africa |
| United Kingdom | Rest of Middle East |
| United States of America | |
| Japan | |
| Canada | |
| South Korea | |
| Australia | |
| Switzerland | |
| Taiwan | |
| Norway | |
| Finland | |

**Supplementary Discussions**

## SD 1. Representing the monetary value of South-North transfers

The net-appropriation or drain of labour from the global South is best understood in terms of potential use-values. However, in addition to calculating the physical scale of the net-appropriated labour, we have also represented it in terms of prevailing Northern wages as a point of reference. Taking this approach, and accounting for skill levels, we find that the wage value of net-appropriated labour was equivalent to €16.9 trillion in 2021. This figure can also be understood as approximating the additional money that Southern workers would earn for labour embodied in their exports to the global North if they were paid equal wages to Northern workers for work of equal skill.

Another way to conceptualise the wage value of the labour drain is to consider that if the North was to maintain its existing levels of consumption without net appropriation, then – all else being equal (in other words, assuming equal labour productivity) – it would require mobilizing an additional 826 billion hours of labour, at the cost of €16.9 trillion in wages. However, if the North has higher levels of productivity, they could maintain their existing levels of consumption in the absence of unequal exchange by mobilizing less than 826 billion hours of additional labour, and therefore with less cost. But this point is moot to the extent that Northern imports from the South cannot be substituted (in the case of products such as coffee, coltan, etc), or to the extent that the North cannot substitute all of the necessary factors of production (such as land and materials), or to the extent that a sufficiently large quantity of additional labour cannot be mobilized, etc.

It is important to note that this approach (regardless of the wage level used to establish the monetary representation of the appropriated labour) does not capture the full scale of value transfer through unequal exchange. Workers produce not only the value they receive as wages, but also the value that is captured as profits. If roughly half of total production is captured as profits (or otherwise not distributed in the form of wages), then the total value of production is double the wage bill. If we use global North figures, where the labour share of GDP was 55.4% in 2021, 826 billion hours of labour would have produced total value equivalent to about €30.5 trillion.

Monetary representations of value transfer through unequal exchange are always to some extent thought experiments. Under prevailing conditions, we know that 826 billion hours of labour mobilized in the global North, with the particular skill profile of the drained quantity in 2021, would produce €30.5 trillion worth of value. It is reasonable to assume that if Southern labour and resources were not cheapened, and if Southern economies had been free to develop their productive capacities, then with a similar quantity of labour they would produce a similar quantity of value. With respect to this counterfactual scenario, the drain of 826 billion hours in 2021 can be said to represent a value transfer of €30.5 trillion.

This result more closely follows Amin's conception of value transfer. Amin calculated value transfer as the difference between the total prices of Southern exports to the North versus the prices those products would have achieved if they were provided by the North, "with the same techniques and so the same productivity."[1] Similarly, the €30.5 trillion figure

[1] Amin, S. *Unequal development: an essay on the social formations of peripheral capitalism*. (Monthly Review Press, 1976).

approximates the additional quantity of money the South would have received for their exports to the North in 2021 if they achieved Northern prices. Of course, as with all monetary representations of value, this figure should be treated with caution. Northern prices are artefacts of the *existing* economy (i.e., its existing balance of power, its existing level of commodification, etc.), including existing dynamics of unequal exchange. In a counterfactual scenario *without* unequal exchange, Northern prices would be different.

**SD 2. On the question of productivity**

This study demonstrates that large North-South wage inequalities and unequal exchange occur even when accounting for sectors and skill levels. It is possible that part of this is due to productivity differences, although in the main text we describe briefly why this is unlikely to substantially explain the extent of unequal exchange.

This question cannot be assessed using conventional productivity metrics (which rely on prices), for reasons we describe in the main text. Andrew Fischer explains:

> "The problem with using value-added as a proxy for productivity is that value-added represents a combination of output (tangible and intangible) and prices/wages. 'Productive' employment in the tertiary sector, for instance, is as much a reflection of wage rates in that sector as any notion of productive output *per se* (for example, what is the output of a lawyer or a bureaucrat?). Hence, the use of value-added as a shorthand for productivity leads to absurd logical implications, such as the suggestion that a barber in the United States is 30 or more times more productive than a barber in India even though they both 'produce' the same number of haircuts per hour (according to the tastes and expectations of their clients), simply because the wage of the barber in the United States is 30 or more times higher. …In other words, much of what we are picking up in most conventional measures of productivity actually amounts to price or wage differences, not actual effort or output, especially in economies that are increasingly based on services."[2]

Physical capital per worker may better help us understand productivity differences, but this data is only available at the country level (i.e., for total production), when what matters for our purposes is the specific sectors and industries involved in producing goods that are traded between North and South. Ultimately, answering the productivity question empirically would require data on *physical output* per hour of labour time, comparing identical steps in the production process of identical products. Unfortunately, such data is not readily available for the range of countries we analyse here. Even if it was, insights regarding comparable products and processes would not be generalizable to the totality of North-South trade, because – as described in the main text (and as Amin, Emmanuel and others have pointed out) – many of the South's exports have no counterpart in Northern production. Where data on physical productivity comparisons *is* available, it demonstrates that productivity inequalities do not explain income inequalities.[3] Indeed, studies show that many export

---

[2] Fischer, A. M. Beware the Fallacy of Productivity Reductionism. *Eur J Dev Res* 23, 521–526 (2011).
[3] Fix, B. The trouble with human capital theory. *Real World Economics Review* 86 (2018).

industries in the global South are as productive, if not more, than Northern firms producing similar products. As Arjun Makhijani writes:

> "The product that export platform countries in the developing World are selling is not merely cheap labour, but highly productive labour. In Singapore McGraw Hill produces in one year an encyclopedia that takes five years to produce in the U.S. ... Mexican metal workers are 40 per cent more productive than U.S. workers, electronics Workers 10 to 15 per cent more productive, and seamstresses produce 30 per cent more sewing per hour than their U.S. counterparts."[4]

The high productivity of Southern labour in these cases is not surprising, given that most Southern export industries use advanced technologies provided by foreign capital. Samir Amin points out that "the exports of the Third World are not, in the main, made up of agricultural products coming from backwards sectors with low productivity."[5] On the contrary, he estimates that even as early as 1966, some three-quarters of the South's exports to the North were produced in "the ultra-modern capitalist sector (oil, mining and primary processing of minerals, modern plantations – like those of United Fruit in Central America, or of Unilever in Africa and Malaya)". It is likely that this proportion has increased over the past several decades as a result of globalization and the rise of global commodity chains.

For trade in services, where the comparability criteria can be met more easily (e.g., IT work, call centers, commercial aviation, tourist hospitality, finance, etc.), there is generally little if any difference in physical output per hour. And yet, as we have shown in this study, large wage inequalities and unequal exchange persist even in this sector. For example, the physical productivity of labour employed the Marriott hotel in Accra, Ghana will be similar to that of the Marriott hotel in Calgary, Canada, but wage inequalities between the two are very large.

Of course, it is likely that some export industries in the global South continue to operate with less productive technologies. However, dependency theorists argue that unequal exchange still occurs in these cases because wage differentials are greater than productivity differentials. According to Samir Amin, in 1966 some 25% of the South's exports were "provided by the backward sectors with low productivity (agricultural produce supplied by peasantries of the traditional type)."[5] Drawing on contemporary agronomic studies, Amin estimated productivity in this sector could be doubled through the application of "modern European techniques." In other words, if productivity alone determined unequal exchange, we could expect a labour exchange ratio of 2:1. But in reality the observed ratio is much larger than this (the present study demonstrates ratios between 10:1 and 20:1). Amin thus concluded that the bulk of unequal exchange could not be accounted for by productivity differences. This is confirmed by a recent study finding that a country's relative technological endowment (as measured by the World Economic Forum's technological adoption index)

---

[4] Makhijani, A. *From Global Capitalism to Economic Justice: An Inquiry into the elimination of systemic poverty, violence and environmental destruction in the world.* Apex Press (1992).

[5] Amin, S. *Unequal development: an essay on the social formations of peripheral capitalism.* (Monthly Review Press, 1976).

does not meaningfully predict the scale of wage inequalities and unequal exchange, which appear to be driven primarily by other factors.[6]

Andrew Fischer has presented Arthur Lewis' explanation for "why tropical produce is so cheap".[2] Lewis wrote: "Take for example the case of sugar. This is an industry in which productivity is extremely high… [with] a rate of growth of productivity which is unparalleled by any other major industry in the world – certainly not by the wheat industry. Nevertheless workers in the sugar industry continue to walk barefooted and to live in shacks, while workers in wheat enjoy among the highest living standards in the world." Lewis argued that national wages are set in the "subsistence sector" rather than in the export sector, so improvements in productivity in the latter do not result in proportionately higher wages. Therefore, the benefits are accumulated by the Northern importers, who are able to buy more of the product for the same price. Lewis noted: "the prices of tropical commercial crops will always permit only subsistence wages until, for a change, capital and knowledge are put at the disposal of the subsistence producers to increase the productivity of tropical food production for home consumption."

Fischer writes: "Later in his life, Lewis (1978:36) similarly predicted that even as developing countries would move into manufacturing exports, these new exports would function in a manner similar to the previous agriculture export commodities, in the sense that increasing productivity would simply reduce the prices of such manufacturing exports. If we are to believe the evidence regularly laid out by UNCTAD, for instance, this prediction appears to have proven true in the three decades since he made this prediction, at least for the huge increase in Southern manufacturing exports that are integrated into international production networks dominated by transnational corporations."[2]

This is consistent with what Emmanuel established, that the poor terms of trade suffered by developing countries cannot be explained by differences in product categories. He is worth quoting at length:

> "The 'worsening of the terms of trade for primacy products' is an optical illusion. It results from a mistaken identification of the exports of the rich countries with the export of manufactured goods and of the exports of the poor countries with the export of primary products. The copper of Zambia or the Congo and the gold of South Africa are no more primary than coal, which was only yesterday one of the chief exports of Great Britain; sugar is about as much "manufactured" as soap or margarine and certainly more "manufactured" than Scotch whisky or the great wines of France; before they are exported, coffee, cocoa, and cotton (especially cotton) have to undergo a machine processing no less considerable, if not more so, than in the case of Swedish or Canadian timber; petroleum necessitates installations just as expensive as steel; bananas and spices are no more primacy than meat or dairy products. And yet the prices of the former decline while those of the latter rise, and the only common characteristic in each case is that they are, respectively, the products of poor countries and the products of rich countries. Textiles were formerly among the pillars of the wealth of the industrialized countries, and Britain's warhorse; since they have become the specialty of poor countries, their prices hardly

[6] Dorninger, C. *et al.* Global patterns of ecologically unequal exchange: Implications for sustainability in the 21st century. *Ecological Economics* 179, 106824 (2021).

suffice to provide a starvation wage for the workers who produce them and an average profit for the capital invested in their production, even where the technique employed is the most up-to-date. Must we suppose that by an amazing coincidence at the same moment when the change of location took place there occurred a reversal in the elasticities of demand? Are there really certain products that are under a curse, so to speak; or is there, for certain reasons that the dogma of immobility of factors prevents us from seeing, a certain category of countries that, whatever they undertake and whatever the produce, always exchange a larger amount of their national labor for a smaller amount of foreign labor?"[7]

Importantly, large North/South wage inequalities and patterns of unequal exchange persist even while industrial production has increased dramatically as a share of the South's total production and exports in the decades since Emmanuel wrote these words. As of 2010, at least 79% of the world's industrial workers live in the South (data from the ILO), and the majority of Southern exports (70%) consist of manufactured goods (data from UNCTAD).[8]

In the main text we noted that in cases where physical productivity differences do exist, this is often because it is more profitable for capital to use cheaper, more labour-intensive methods than to invest in modern equipment – especially in cases where state investment in technological development has been curtailed by structural adjustment programmes, or where patents prevent affordable access to necessary technologies – precisely because Southern wages are maintained at artificially low levels. This arrangement benefits Northern consumers with cheaper goods and benefits Northern capital with increased surplus. In such cases, the use of labour-intensive methods facilitates value transfer and should be understood as constituting unequal exchange.[9] Under these conditions, the South is compelled to allocate more labour to production for international trade than would be required if technology was deployed more rationally and fairly, thus draining – and wasting – a crucial productive capacity that could otherwise be allocated toward producing goods and services necessary for local well-being and development.

**SD 3. Comparison to previous work**

The results of this study show that the North's net appropriation of embodied labor from the global South is substantially larger than indicated in a previous study based on the EEMRIO model EORA, through 2015.[10] This is because the EORA model systematically understates labour flows in the world economy compared to other EEMRIO models. EORA shows a global total of 2.38 billion person-years of labour in 2015, while more recent models indicate much higher values: Gloria, an update to EORA, shows 3.13 billion, and EXIOBASE shows 3.55 billion in the same year. EORA indicated that the global North (defined according to the same country

[7] Emmanuel, A. *Unequal exchange; a study of the imperialism of trade*. (Monthly Review Press, 1972).

[8] Smith, J. C. *Imperialism in the twenty-first century: globalization, super-exploitation, and capitalism's final crisis*. (Monthly Review Press, 2016)

[9] Sullivan, D. 'Unequal Exchange and the question of productivity' (in submission, 2024).

[10] Hickel, J., Dorninger, C., Wieland, H. & Suwandi, I. Imperialist appropriation in the world economy: Drain from the global South through unequal exchange, 1990–2015. *Global Environmental Change* 73, 102467 (2022)

grouping we use in this paper) net-appropriated 188 million person-years from the global South in 2015, while EXIOBASE indicates roughly 78% more in the same year.

The present research shows that the EORA-based study also underestimates the monetary value of the North's net appropriation of labour. The EORA study was unable to measure the wage value of drain directly, as wage data was not available; instead, the drain was calculated using the prices of traded goods estimated by assuming an equal share of labour, land, energy and materials in TiVA (trade in value added); in other words, with each flow comprising 25%. With this method, the drain of labour was estimated at $5.1 trillion in 2015 in Northern prices, with land, energy and materials comprising another $5.7 trillion in that year (for a total drain of $10.8 trillion). EXIOBASE indicates that labour comprises much more than 25% of TiVA (around 50%). The present study using EXIOBASE shows that the monetary value of the labour drain was €12.8 trillion in 2015 in Northern prices. The total value of drain would therefore be roughly double this.

In sum, the EORA-based study underestimates the drain of labour both in physical and wage-value terms. The present study supersedes the previous work with a more up-to-date model and direct data on wages, delivering more specific results. It is worth noting, however, that while the two studies yield different results when it comes to the scale of the labour drain, the results are similar in terms of trends over time. Both studies show an increasing drain until 2005, followed by decline and stabilization (which is also confirmed by results based on other methods[11]).

[11] Hickel, J., Sullivan, D. & Zoomkawala, H. Plunder in the Post-Colonial Era: Quantifying Drain from the Global South Through Unequal Exchange, 1960–2018. *New Political Economy* 1–18 (2021)
