# Supplementary material for: Unequal exchange of labour in the world economy
Source: Nat Commun. 2024 Jul 29;15:6298. doi: 10.1038/s41467-024-49687-y (PMC11286830; doi:10.1038/s41467-024-49687-y)
Supplement: Supplementary file 3 — Description of Additional Supplementary Files [file 41467_2024_49687_MOESM3_ESM.pdf]

Description of Additional Supplementary Files

File Name: Supplementary Data 1

Description: Allocation of products to sectors, based on recommended sectoral aggregation of industry tables in EXIOBASE
